# Incobotulinumtoxin A and Yoga-like Isometric Exercise in Adolescent Idiopathic Lumbar Scoliosis—A Randomized Pilot Study

**DOI:** 10.3390/muscles3010004

**Published:** 2024-02-01

**Authors:** Loren Fishman

**Affiliations:** Department of Rehabilitation and Regenerative Medicine, Columbia University Medical School, New York, NY 10032, USA; loren@sciatica.org

**Keywords:** adolescent idiopathic scoliosis, botulinum toxin, yoga

## Abstract

Background: Approximately 90% of scoliosis cases are adolescent-idiopathic (AIS). From the first appearance of scoliosis at 10–14 years of age until the age of 18, the spine is most vulnerable to deterioration; young, growing people are most susceptible to the worsening of one or more scoliotic curves. An effective non-surgical means of remediation would be welcome. Design: This was a randomized, controlled, two-arm study assessing the safety and efficacy of combining incobotulinum injections with yoga to reverse lumbar and thoracolumbar AIS. Methods: In a private clinic setting, non-pregnant, healthy 12–18 year-olds were either taught a symmetrical “placebo” yoga pose (control sub-group 1), performed the side plank (Vasisthasana) three times daily with a placebo injection (control sub-group 2) or performed the three-times-daily side plank with a botulinum injection (intervention group 3). Injection: For the injection, 33 IU of incobotulinumtoxin type A (Xeomin) was injected into the concave-side lumbar paraspinals and quadratus lumborum at L2–3 and the psoas muscle at L3–4, or participants were injected similarly with a placebo. Randomization was achieved using random.org. Objective: The objective was to determine whether the treatment of muscular asymmetry with botulinum toxin injections and side planks is safe and effective in AIS. Results/Outcome: Eleven intervention and thirteen placebo patients (Groups 1 + 2), who were 12–18 years old, completed the three-month study. Mean daily side plank time = 165 s. The mean initial lumbar curvature was 36.9 degrees (SD 14.36), (*p* < 0.0001); the mean Group 3 curvature at 3 weeks was 29.5 degrees (SD 14.23) (*p* < 0.0001); and the mean Group 3 curvature at 3 months was 26.0 degrees (SD 12.81). Onset vs. 3-month value: *p* < 0.0001. Harms were limited to one patient in Group 2 and one in Group 3, who complained of transient shoulder pain and supported themselves temporarily on their forearm instead of the palm of the extended hand. Conclusion: Muscle strength asymmetry appears to be relevant to AIS treatment. Incobotulinum injections combined with side planks performed with the convex side downward may be more effective in reversing lumbar AIS than placebo exercises or side planks and placebo injections.

## 1. Introduction

Scoliosis is a three-dimensional curve composed of side-to-side curve(s) and posterior rotation toward the convex side of one of the curves. It is seen in approximately 2% of Americans and 2% of the population worldwide [1]. Given that Earth’s human population is approximately 8 billion, if these percentages run true, then 80 to 160 million people suffer from AIS worldwide. Since the advent of antibiotics for tuberculosis and the Salk vaccine for polio, approximately 85% of scoliosis is adolescent-idiopathic (AIS) [2]. Although females are more likely than males to acquire the condition, the ratio changes from 1.4:1 in curves of 10–20 degrees to 7.2:1 in curves above 40 degrees [2]. Although many asymmetric sports such as tennis and baseball appear to have no relation to scoliosis, dancers are at significantly greater risk of it [3]. It is uncertain whether this is due to ligamentous and bone changes or to the greater flexibility of dancers, enabling the asymmetric distribution of the spinal muscles’ strength to curve the spine more.

When a Cobb angle measurement of a curve exceeds 25 degrees, braces are often implemented to deter progression, since this is the level at which most studies report the highest rates of progression [4,5,6,7]. However, several other systems may make different determinations. The Rigo–Cheneau classification, which articulates its own guidelines for implementation, designs and crafts braces which may depart somewhat from the 25-degree demarcation [8]. It coordinates brace design with fabrication, using its own principles of correction, and thus has control over the relationship between when to prescribe and what is furnished. The soft SpineCor brace has its own system as well, with its own system of internal consistency [9].

Braces are not generally expected to diminish curvature, but rather reduce curve progression [10]. Nevertheless, the SpineCor and Lyons braces have been shown to have corrective capacities [11,12]. When the goals of bracing were polled among authorities in the field, aesthetics, quality of life, disability, back pain and psychological well-being were found to be the most important goals, in that order [13]. These goals are naturally promoted through curve correction as well. The same group judged the evidence in favor of bracing to be stronger than the evidence for any other conservative modality, with scoliosis-specific exercises second [13]. Nevertheless, discomfort, high-school embarrassment, lowered self-esteem, body image [14] and consequent issues of compliance are relevant, and questions have been raised of whether core stabilization or scoliosis-specific exercises, and even the use of a second orthotic or insoles, improve braces’ efficacy [13,15]. Other conservative methods, such as the Schroth, and chiropractic systems, such as Pettibone and Clear, have mixed reports regarding efficacy, and physical therapeutic exercise programs are also currently being tested [16,17,18,19]. Greater clarity on ancillary treatments with braces is desirable, especially on the underlying principles that can guide therapeutic decisions.

Typically, it is only when curves intensify beyond 45 degrees that surgical intervention is considered. Surgery has dominated the field of scoliosis since it rightfully has been recognized as the most reliable and effective remediation. The natural history of AIS suggests 0.4 to 2.2 degrees of annual progression, depending upon age, Risser number and curve; teenagers’ spines are capable of much greater change, and great person-to-person variation exists [4,5,6]. When visiting their surgeon, young patients have an X-ray taken, and they and their families are told of the 45-degree threshold for surgery. As a consequence, with or without braces, parents and their children with AIS are relegated to the passive role of “watchful waiting” unless and until curves reach 45 degrees.

Although genetic, anatomical and neuroanatomical correlates of AIS have been discovered [20,21,22,23], promising physiotherapeutic work to effectively stabilize and reverse scoliosis awaits high-quality studies that confirm it [24]. A reliable, innocuous and readily available conservative means of reversing scoliosis curves would be desirable. Such a method would be particularly valuable since, unlike major surgery, it could be instituted in patients with much smaller curves, when treatment would commence earlier and likely would not last as long. 

Previous work suggests electrophysiological and hormonal muscular asymmetries are at work in AIS [25,26], supporting the possibility that muscular imbalance may be a relevant factor in its pathogenesis. We tested this hypothesis by utilizing botulinum toxin type A, incobotulinum, a medication that temporarily weakens muscles, on the convex side. It has few other effects when injected intramuscularly.

The hypothesis that muscular imbalance is important in AIS is also supported by a study finding that the Schroth method, a muscle-oriented treatment, significantly improved curves [19]. Further, a single yoga pose, the side plank, performed with the convex side of lumbar curves held inferiorly, was found to be helpful in AIS in multiple studies [27,28,29]. A randomized, controlled repeat of this method found it ineffective [30], but close reading of that study reveals that unfortunately, the randomization of the intervention group was such that not a single patient with a lumbar curve was actually included in it [31]. In the current study, we used the side plank to strengthen the weaker (convex) side and added botulinum toxin type A injections of the contralateral paraspinal, quadratus lumborum and psoas muscles to temporarily weaken the stronger (concave) side. Bracing of patients was not permitted during the test period to avoid possibly confounding factors. Testing the validity of the muscular imbalance hypothesis, the primary and secondary objectives of this study were to assess the benefits and the harms [32] of combining incobotulinum A injections with yoga to reverse lumbar and thoracolumbar AIS [28,29,30].

The current study has been approved by the Chesapeake IRB (now Advarra) and the FDA, since this use of botulinum toxin is virtually new in the United States; one other institution is studying it in a similar context [33]. The current study was made public on Clinical Trials.org NCT04922983 on 17 July 2021, and was accessed on that day.

Recruitment began 1 July 2021, and is reported because the results seem to us important enough to make them public as soon as possible.

## 2. Results

**Figure 1:**
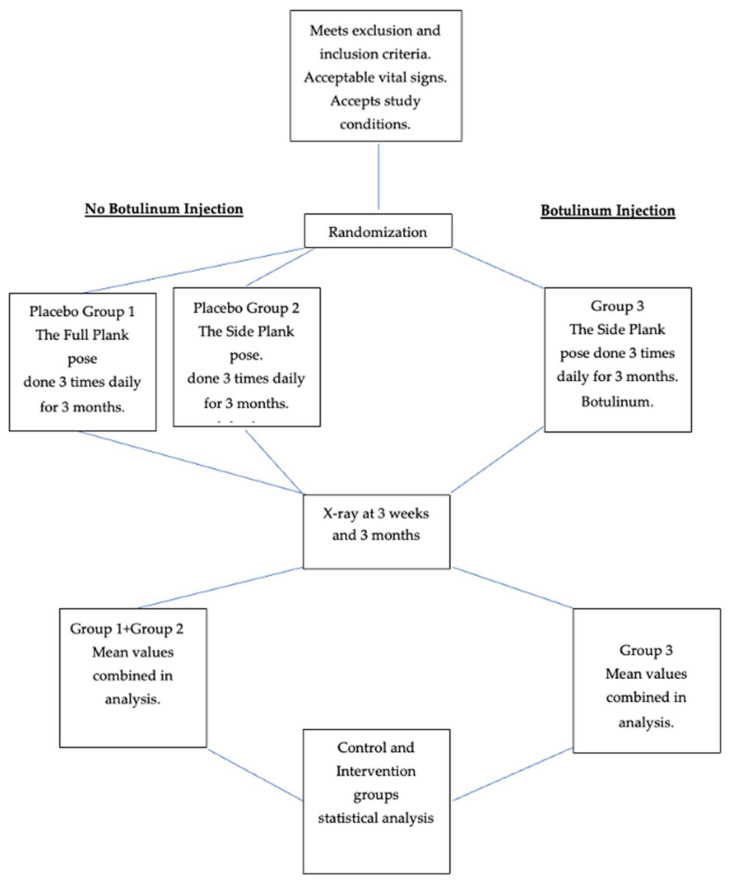
Flow chart of study.

Groups 1 and 2 made up a significant control group of 6 + 5 = 11 patients, with 1 male in Group 1. See Figure 1. Thirteen patients, including four males, completed the protocol in Group 3. Mean age of controls and intervention patients: Group 1: 16.8 (S.D. 1.3); Group 2: 14.7 (2.1); Groups 1 + 2; 15.3 (2.3); Group 3: 15.9 (1.75). Mean weight of controls and intervention groups: Group 1: 123.6 lb. (18.25); Group 2: 116 lb. (15.92); Group (1 + 2): 121.4 lb. (25.1); Group 3: 123.85 lb. (11.95). See Table 1. 

There was one dropout in Group 1, and two dropouts in both Groups 2 and 3 that were non-compliant at second or third X-rays. Three prospective patients experienced injection anxiety after randomization but before any treatment was initiated, and therefore were not treated or included in the study. There were no reported injuries from the yoga pose in any group beyond a few days of sore shoulder and forearm muscles: one patient in Group 2 and one in Group 3 had transient complaints of this nature. These two patients continued the side planks on their forearms, but they did not otherwise alter their yoga routines. There were no changes in vital signs or later side-effects after administration of incobotulinum. With rarely missed days, all patients reported performing the side plank or full plank at least twice daily beginning at a mean 35 s per side plank, with a mean initial cumulative reported dose of 85 s daily and ending at a mean 70 s per side plank after 3 months, with a mean cumulative dose of 165 s daily, during the three month period. Most participants performed the multiple side planks successively in the morning. 

Mean lumbar scoliosis at study onset: Group (1 + 2) 37.91 degrees (S.D. 12.78); range: 25–60 degrees. Group 3: 41.85 degrees (S.D. 16.2); range: 18–69 degrees. Mean 3-week curve Cobb measurements were Group (1 + 2): 37.73 (11.38); Group 3: 33.15 (13.95). Cobb measurements at 3 months were Group (1 + 2): 35.82 (11.12) and Group 3: 29.46 degrees (12.59). See Table 2 and Table 3.

**Figure 2:**
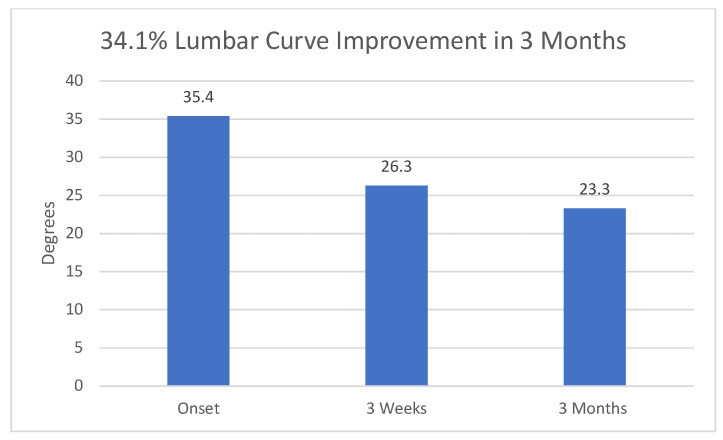
Overall reduction in Cobb angle in Group 3. 95% confidence intervals at onset, 3 weeks and 3 months were 9.795, 8.434, and 7.612, respectively.

Significant differences appeared between Groups 1 and 3 at three weeks (*p* = 0.0015) and at 3 months (*p* = 0.0005); between Groups 2 and 3 at 3 months (*p* = 0.0365); and between Groups (1 + 2) and Group 3 at 3 weeks (*p* = 0.0031), at 3 months (*p* = 0.0002) and between 3 weeks and 3 months (*p* = 0.0328). See Table 1, Table 2 and Table 3 and Figure 2.

Apart from the transiently sore shoulders and forearms mentioned above, no harms were seen in any participants, although they were rigorously sought along SOSORT guidelines [13].

## 3. Design

This is a randomized, controlled study, with a split control group: one sub-group received a “placebo” yoga pose only, while the second received the intervention yoga pose and normal saline (placebo) injections. The intervention group received both the interventional yoga pose and botulinum injections. Recruitment began on 1 July 2021.

### 3.1. Eligibility

#### 3.1.1. Inclusion Criteria

Age 12–18 years.
Lumbar or thoracolumbar curve of 25 degrees or more.
Willingness to perform one yoga pose for as long as possible three times daily for three months.
Parental or guardian agreement.

#### 3.1.2. Exclusion Criteria

Neuromuscular or musculoskeletal disease, e.g., cerebral palsy, Guillain–Barre syndrome, Marfan’s syndrome.
Current use of brace.
Previous spinal surgery.
Previous exposure to botulinum toxin type A.
Positive pregnancy test.

#### 3.1.3. Particulars of the Study

The study was conducted in private offices in Manhattan, New York, USA.

The study accepted non-pregnant applicants 12 years–18 years of age who had at least 25-degree lumbar or thoracolumbar curves on X-rays completed less than 6 months before their visits. Control patients were given the regular yoga pose, the plank, that consists of a symmetrical two-handed suspension of the upper body with extended elbows and lower body suspended on dorsiflexed feet. Intervention group participants were given the side plank yoga pose (*Vasisthasana)* in which the body is supported by one extended arm with the torso’s coronal plane perpendicular to the floor and the lower body weight supported by the lateral foot of the downward side. Instructions to both placebo and interventional participants were that the poses were to be performed three times daily for as long as possible each time. Intervention poses were performed with the concave lumbar or thoracolumbar curve downward. Intervention group patients were also injected with 100 U of botulinum toxin type A divided into three equal doses of 33.3 IU. All incobotulinum injections were applied to the concave side of the curve: one dose at the paraspinal musculature opposite the lumbar curve’s inflexion point, generally L2-3; one into the quadratus lumborum opposite L2-3; and the third into the psoas muscle injected from a posterior approach approximately 7 cm lateral to the spine at L4. Paraspinal and quadratus lumborum injections were performed with 1.5 inch inoject needles; the psoas injection was performed with a 7 inch inoject needle; all injections were conducted under EMG guidance. Patients’ vital signs and weight were tested before the injections and again (except for weight) 15 min after the injections. Patients repeated their scoliosis X-rays at 3 weeks and 3 months. EOS technology was used whenever possible to minimize exposure to radiation. Checks on participants’ compliance with the three-times-daily side plank regimen were attempted by telephone and email.

Power calculations based on previous papers [23,24,25] yielded 10 subjects in the control group and ten subjects in the study group and the placebo groups, where alpha = 0.05 and (1 − beta) = 80%, (10 subjects per group). Randomization was conducted using random.org as patients qualified for the study presented in the office. There was no blocking.

The medical assistant enrolled the patients; the office manager generated the randomized treatment group. The medical assistant prepared the syringe with non-preservative normal saline or incobotulinum plus 1 cc of preservative-free normal saline, both colorless liquids. The participants, care providers and radiologists performing the initial and subsequent scoliosis X-rays and measuring Cobb angles were all blinded regarding group assignment. Apart from Group 1, which performed the two-handed ‘placebo’ yoga pose, and which by necessity was different in appearance from the intervention yoga pose, and the fact that this group had no injection, all procedures were indistinguishable to participants, care givers and radiologists.

One-tailed *t*-tests were used to test the hypotheses, since only curve improvement was sought. 

## 4. Discussion

The data, results and implications of this small study must be regarded with caution. However, “When a new treatment is introduced, it is not possible to wait years (end of therapy) before verifying its utility” [32]. We have viewed the spine as a tensegrity structure, a concept of the architect Buckminister Fuller which embraces configurations known for their strength and dynamic response to load [34]. Tensegrity structures are not held together by nails or rivets but by tensions between their parts. Tent poles, Roman arches and radio antennae with their supporting cables are examples. The solar system and the Bohr atom are somewhat extended examples, with gravity, electrical charges and centrifugal force providing the invisible tethers that generate tension and retain the structures’ integrity. The spine may be seen as such a structure, but unlike the static edifices of architecture, the spine is held together by the quite variable tensions of the muscles that surround it. Seen this way, pervasive muscular asymmetry could be a major aspect of scoliosis. 

Throughout the phylum *Chordata*, the spinal cord and the notochord are composed of many segments or metameres. These elementary units are interrelated in their control and in their movements and comprise a basic defining characteristic of the phylum. The spinal cord and its attendant ligaments, and, critically, its muscular attachments, always allow for movement in all three planes, although this differs greatly from, e.g., cattle to humankind. In the turtle, it is the ribs that have coalesced to form the shell; inside it is a segmented creature with a flexible spine. In the course of evolution, there are, to the author’s knowledge, no cases in which a single bone forms the spine as the femur forms the sole support in the thigh. Throughout the phylum, from reptiles to humankind, the spine is always firm, but flexible in its multiple vertebrae in order to aid our various bending, twisting, liftings and inclinations, yet the prominent surgeries of our day fuse the spine, rendering portions of it inelastic in a way nature has never allowed. An alternative therapy that repairs the spine without fixing it in a set conformation would be advantageous.

Significant improvement in Cobb angles at three weeks post-injection in Groups 1 vs. 3, as well as in Groups (1 + 2) vs. 3, is not surprising but does support the hypothesis that some AIS is due at least in part to muscular imbalance, and that at least one of these three muscles: the paraspinal musculature, the quadratus lumborum or the psoas is involved in that asymmetry. Less obvious is how to explain the further improvement at three months, given that botulinum toxin type A is essentially inactive after two months following injection or even earlier. Three factors may help explain this:

(1) Although inactive after two months, longer-term reduction in muscle tension is seen in botulinum toxin’s cosmetic and dental uses [35,36,37,38,39].
(2) The botulinum weakens the strong (concave) side of the lumbar curve, enabling the actin and myosin fibers of the weak (convex) side to slide further together, increasing the number of cross bridges, and proportionately increasing their power to contract [40], in addition to the continual strengthening the yoga pose provides during the latter part of the three-month period. These considerations must be viewed as hypotheses at this point, needing further confirmation or contradiction.
(3) Three-times-daily practice of the side plank yoga pose alone, held for as long as possible once daily, has been shown [27,28,29] to reverse lumbar curves due to AIS, thus increasing its strengthening effect on muscles of the convex side of the lumbar curve during the three-month period.

Of note is the insignificance of the 3-week vs. 3-month values’ changes in the placebo groups (1 and 2), suggesting that the effect of the botulinum toxin type A injections was a significant factor in the patients’ recoveries. See Table 2 and Table 3. In studies showing the efficacy of the yoga pose alone, 5–12-month inter-X-ray periods were used [27,28,29].

The adolescent idiopathic scoliotic spine is vulnerable to increasingly severe deepening of its curve. This is evidenced in the increasing Cobb angles seen in some patients and suggests that the actual advantage of the botulinum-plus-side-plank program may even be greater than those seen in this study of adolescents. This tendency of AIS to worsen dramatically in the teen years may to some extent obscure the actual benefit that intervention group patients received regarding the corrective influence of the yoga plus botulinum injections.

**Figure 3:**
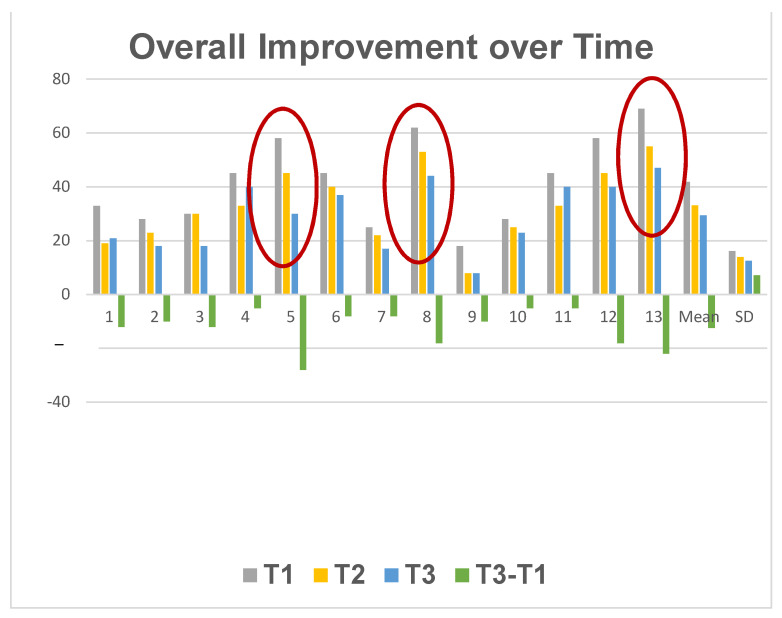
Individual patients’ course in the three months following inception of the side plank and incobotulinum injection. T1 = Cobb angle at time of injection. T2 = Cobb angle 3 weeks post-injection. T3 = Cobb angle 3 months post-injection. Cases showing greatest improvement are encircled.

We found that the larger curves derived larger benefits from the botulinum-plus-isometric-exercise regimen, suggesting that muscular imbalance is a basic element in at least some cases of AIS. If there were just a constant improvement seen after botulinum injection, it would suggest that although muscular imbalance was indeed a factor, it was not one to which Cobb angle was sensitive, and thus was not a major factor in curve pathogenesis or size. See Figure 3.

If the efficacy of this method is borne out in larger studies, it is sufficiently innocuous, low-cost and readily available to enable young people and their parents to treat lumbar and thoracolumbar AIS as it develops, and before it reaches anatomically and socially significant levels.

In addition, a new botulinum formulation, Doxxibotulinum toxin, is alleged to last 6 months in its active form. This might reduce the need for repeated injections if indeed they prove to be necessary.

## 5. Limitations of the Study

(1) Although it reached statistical significance, this randomized controlled study is based on a small sample. Larger, randomized controlled trials are clearly necessary to demonstrate the efficacy of the botulinum-plus-yoga treatment more reliably.
(2) A single blinded radiological opinion was utilized throughout this study. A second and even a third blinded radiologist (for non-unanimous assessments) would improve the objectivity in these studies.
(3) The opposite limitation is also present: the ranges of the patients’ Risser numbers, ages and curve sizes are too large. Some researchers find that a combination of bracing and exercise is differentially effective in AIS at different Risser numbers and this type of variability may apply to botulinum as well [4,5,6].
(4) Studies have found that bracing plus exercise substantially improve curves in AIS [41]. Studies using bracing and exercise, including the side plank and botulinum toxin injections, might further advance and enhance conservative treatment.
(5) Further study design can also raise the level of objectivity regarding harms, e.g., by measuring activities of daily living [42]. More specific considerations mentioned by leaders in the field may also be relevant, including aesthetics, quality of life, disability, back pain, psychological well-being, self-esteem, body image and embarrassment in high school [13,14].
(6) Longer follow-up is also necessary to demonstrate the value of the treatment. Two- or three-year follow-up or more would be desirable.
(7) This study injected the minimal effective doses of botulinum. Dosages up to 1.67 times greater are patently safe [43]. It is possible that a proportionately greater effect would be seen with larger doses of incobotulinum. This study does not answer that important question.
(8) One may additionally ask about whether the most relevant muscles have been treated. The iliocostalis, longissimus, semispinalis and spinalis muscles, as well as the external and internal intercostals and obliques, the superior and inferior serratus posterior, the subcostal, the quadratus lumborum, the latissimus dorsi and trapezius, the transversus abdominis, the rectus abdominis and the diaphragm itself might all function to laterally flex and/or rotate the spine. These muscles should be studied, both with different yoga poses and other types of exertion vis à vis strengthening them, and for appropriate dosages of botulinum toxin for weaking their contralateral counterparts.

## 6. Conclusions

Muscular imbalance appears to play a part in the pathogenesis and longevity of adolescent idiopathic lumbar scoliosis. The side plank and botulinum toxin type A injections may be more effective in reversing lumbar AIS than a placebo yoga pose or the side plank along with a placebo injection.

## Figures and Tables

**Table 1:** Demographic information for Groups 1, 2 and 3.

Group	#	M *	Age (SD)	Weight (SD)	Risser #	T1	T2	T3
Group 1	6	1	16.8 (1.3)	123.6 (18.25)	3.25 (1.26)	35.75 (17.25)	37 (15.32)	37.5 (13.89)
Group 2	5	0	14.7 (2.1)	116 (15.92)	3.7 (1.27)	38 (13.04)	33.40 (11.46)	33.40 (12.16)
Group 3	13	4	15.9 (1.75)	123.85 (11.95)	3.6 (0.96)	41.9 (16.2)	33.15 (13.95)	29.46 (12.59)
Group 1 + 2	11	1	15.3 (2.2)	121.4 (25.1)	3.4 (1.24)	37.91 (12.78)	35.73 (11.38)	35.82 (11.12)

* M = Male, SD = Standard deviation. # = Number. T1 = Cobb measurement of lumbar or thoracolumbar curve of X-ray at time of visit. T2 = Cobb measurement of lumbar or thoracolumbar curve of X-ray 3 weeks following visit. T3 = Cobb measurement of lumbar or thoracolumbar curve of X-ray 3 months following visit.

**Table 2:** Intervention group analysis. Mean lumbar Cobb angles at onset = T1, three weeks = T2 and three months = T3.

Group	T1	T2	T2-T1	T3	T3-T2	T3-T1
1	35.75 (17.25)	37 (15.34)	1.25	37.5 (13.89)	0.5	1.75
2	35.25 (13.28)	33.40 (12.46)	7.25	33.16	0	−4.6
1 + 2	37.92 (12.78)	35.73 (11.38)	−2.19	35.88 (11.12)	0.15	−2.4
3	41.85 (16.20)	33.15 (13.95)	−8.7	29.46 (12.59)	−3.69	−12.39
		Mean values				
		( ) = Standard deviation				

**Table 3:** Comparison of Groups 1, 2, (1 + 2) and 3.

Groups	Delta	t-Value	*p*	df	Confid Levels	S.E.
Groups 1 vs. 2						
T2-T1	−5.9	2.0114	0.0971	8	−0.77 to 11.27	2.61
T3-T1	1.75	0.4093	0.6867	16	−8.75 to 12.73	5.108
T3-T2	0.5	0.1832	0.8607	6	−28.71 to24.71	10.92
Groups 1 vs. 3						
T2-T1	−6.35	3.865	0.0015	15	6.56 to 22.71	3.786
T3-T1	−10	4.4055	0.0005	15	5.91 to 16.98	2.597
T3-T2	−4.2	1.2178	0.2421	16	−3.15 to 11.53	3.443
Groups 2 vs. 3						
T2-T1	−5.9	2.0147	0.0611	16	−0.271 to 10.66	2.577
T3-T1	−7.76	2.2827	0.0365	16	−0.54 to 14.63	3.323
T3-T2	−3.7	1.2178	0.2421	15	−3.15 to 11.53	3.443
Groups 1 + 2 vs. 3						
T2-T1	−6.5	3.3096	0.0031	23	−10.31 to −2.38	2.039
T3-T1	−10.4	4.4393	0.0002	23	−8.57 to 12.75	2.358
T3-T2	−3.85	2.2057	0.0382	22	0.24 to 7.94	1.855

Delta = Difference in changes in lumbar Cobb angle between first listed and second listed time in control group (1 + 2) vs. intervention group (3), and between control sub-groups (1 vs. 2). Df = degrees of freedom. Confid. levels = 0.95% confidence level. S.E. = standard error. T1 = Entry into study and time of injection for Groups 2 and 3. T2 = Three weeks post study entry. T3 = Three months post study entry.

## Data Availability

The data for this study can be found at ac@columbia.edu (accessed on 14 January 2014).
